# Endothelial Function in a Mouse Model of Myeloperoxidase Deficiency

**DOI:** 10.1155/2014/128046

**Published:** 2014-02-23

**Authors:** Veronika Golubinskaya, Ulla Brandt-Eliasson, Li-Ming Gan, Martin Kjerrulf, Holger Nilsson

**Affiliations:** ^1^Department of Physiology, Institute of Neuroscience and Physiology, Sahlgrenska Academy, University of Gothenburg, P.O. Box 432, 405 30 Gothenburg, Sweden; ^2^Department of Molecular Pharmacology, AstraZeneca R&D, Pepparedsleden 1, 431 83 Mölndal, Sweden

## Abstract

Myeloperoxidase (MPO) activity is suggested to reduce the function of vascular nitric oxide, thereby contributing to endothelial dysfunction, although data in rodents are inconclusive. We examined vascular contractile and relaxant responses in MPO-deficient (MPO^−/−^) and wild-type mice to investigate the role for myeloperoxidase in the development of endothelial dysfunction. Carotid and saphenous arteries were taken from 8-month-old mice and studied in a myograph. Responses of carotid arteries to phenylephrine, high potassium, or acetylcholine (Ach) were statistically not different from controls. Treatment with lipopolysaccharide (LPS; to enhance endothelial dysfunction) reduced responses to Ach in MPO^−/−^ but did not affect responses in wild-type. In response to high concentrations of Ach, carotid arteries responded with transient contractions, which were not different between the groups and not affected by LPS treatment. Saphenous arteries from MPO^−/−^ had smaller normalized diameters and developed less contractile force. Vessels from MPO^−/−^ were less sensitive to Ach than controls. These data suggest that mature MPO-deficient mice do not show enhanced endothelial function compared to wild-type mice, even when provoked with LPS treatment. The EDHF response appears to be reduced in MPO deficiency.

## 1. Introduction

Endothelium-dependent vasodilatation in response to, for example, elevations of blood flow is considered a hallmark of normal vascular function [1]. Reduced vasodilator capacity—endothelial dysfunction—has been associated with cardiovascular disease, in particular atherosclerosis [2]. Although atherosclerosis affects predominantly the large arteries, endothelial dysfunction is probably a general feature of the endothelium in most parts of the vasculature of the atherosclerotic patient [3].

Recently, attention has focused on the enzyme myeloperoxidase (MPO) as a possible contributor to endothelial dysfunction [4]. MPO catalyses the conversion of hydrogen peroxide to hypochlorous acid. This product may react with nitric oxide, creating peroxynitrite, which has detrimental effects on effector cell function. Hypochlorous acid also chlorinates arginine, thus consuming the substrate for nitric oxide synthase. MPO may also directly catalyse the elimination of nitric oxide. Furthermore, MPO may also increase oxidative stress and oxidise [5, 6] or carbamylate [7] lipoproteins. In these ways, myeloperoxidase activity may directly and indirectly antagonise endothelial function.

Myeloperoxidase is normally expressed in leukocytes, primarily in neutrophils, from which it can be released into the blood stream [8]. Its function in leukocytes is primarily as an antibacterial agent, producing hypochlorous acid to reduce bacterial activity. In humans, myeloperoxidase from the blood stream has been shown to be taken up by endothelial cells as well as traversing the endothelium to become localised in the arterial wall, probably adsorbed to glycosaminoglycans on the endothelial surface and to fibronectin in the subendothelial extracellular space [9]. It has been demonstrated that intravascular heparin may release significant amounts of MPO from tissues into the blood [10]. The amount released by heparin is greater in patients with coronary artery disease [10], consistent with an inflammatory change in the artery wall causing an accumulation of MPO there.

Myeloperoxidase activity has been suggested to be associated with risk for human cardiovascular disease. It could therefore be assumed that MPO deficiency (a not very rare condition in humans) might be beneficial by lowering the tendency to develop atherosclerosis, although this has not been studied directly in humans. Mouse models of MPO deficiency exist [11], but data from these animals are conflicting. Eiserich et al. [11] reported that young MPO knockout mice have reduced endothelial dysfunction when challenged with lipopolysaccharide. Brennan et al. [12], on the other hand, found increased atherosclerosis in LDL knockout mice on a high-fat diet in which the bone marrow had been repopulated with MPO^−/−^ cells after irradiation. The reason for the latter unexpected result is not clear but may relate either to species differences in pathogenesis of atherosclerosis or in other ways to the complexity of the model. We therefore decided to examine MPO^−/−^ mice further with respect to endothelial function at older age, to determine whether long-standing lack of MPO may enhance or reduce endothelial function. We chose to study the carotid artery, where endothelium-dependent relaxation is largely due to NO, and the saphenous artery, a smaller peripheral vessel, where endothelium-dependent relaxation is partly dependent also on EDHF.

## 2. Methods

All experiments were approved by the Gothenburg Ethical Committee on Animal Research. Female MPO^−/−^ mice on a C57BL/6 background (generously provided by Dr. S. Baldus, University of Düsseldorf, Germany) and wild-type C57BL/6 control mice aged approximately 8 months were used. A total of 12 knockout and 12 control animals were maintained on standard rat chow (R3, Lantmännen, Sweden). Flow cytometry was used to verify the absence of MPO expression in neutrophils of MPO knockout mice. Seven animals from each group were injected with lipopolysaccharide (LPS) intraperitoneally, 2 mg/kg, 18 hours before the experiment.

### 2.1. Vascular Preparation

Animals (either untreated or LPS-treated) were euthanized and the right common carotid artery and the right saphenous artery were isolated and rapidly transferred to cold physiological salt solution (PSS; see composition below). Vessels were carefully cleaned from surrounding tissue. The middle part of the carotid artery was divided into two equal parts, as was the middle part of the saphenous artery. The artery segments were transferred to a Mulvany-Halpern type wire myograph (model 600A, Danish Myo Technology, Aarhus, Denmark).

After mounting in the myograph, vessels were equilibrated at 37°C for 30 min. Then the normalization procedure according to Mulvany and Halpern [13] was performed, in which the vessels' circumferences were adjusted to where they can be assumed to develop maximal tension, after which they were equilibrated for another 10–15 min.

A sample of each experiment is given in Figure 3 to illustrate the experimental protocols used. Vessels were then activated 4 times, each for 2 minutes with 5 min intervals between activations (once in high-potassium solution alone, once in phenylephrine (PE) 3 *μ*M alone, once in PE 3 *μ*M in high-potassium solution, and finally again in high-potassium solution alone). The response to the contractions in PE 3 *μ*M in high-potassium solution was taken as the maximal contractile response.

Vessels were then subjected to one of two protocols: one to determine the total response to acetylcholine (Ach) and one where the NO- and prostaglandin-dependent parts of this response were inhibited; the remaining response was defined as EDH-like (similar to what would be expected from endothelium-dependent hyperpolarisation). Thus, one vessel of each type (carotid or saphenous) from each animal was activated with 1 *μ*M noradrenaline (NA) and when contraction approached a plateau, increasing concentrations of Ach were added, each time increasing the Ach concentration a half-log unit over the range 1 nM–10 *μ*M. At the end of the concentration-response experiment, and in the continued presence of 10 *μ*M Ach, 300 *μ*M L-NAME was added. After 20 min, 5 *μ*M indomethacin was added and after another 20 min, 10 *μ*M nitroprusside was added. When relaxation to nitroprusside had reached its maximum, the bath solution was replaced by calcium-free solution without agonists, but still with 10 *μ*M nitroprusside, to determine maximal relaxation.

In the same experiment another part of the vessel of each type from the same animal was activated in parallel following a similar protocol, although L-NAME and indomethacin were added before the concentration-response determination to Ach, in order to determine the EDH-like part of the response to Ach.

Responses were expressed in percent contraction where 100 percent was set to the maximal precontraction level in response to 1 *μ*M phenylephrine.

Vascular responses were recorded as active force (*F*) and in further analysis were estimated as active tension (*T* = *F*/2*l*, where *l* is length of the vessel segment).

### 2.2. Preparation of Peripheral Blood Leukocytes (PBL) and Flow Cytometry

One hundred *μ*L of blood was drawn from the tail veins of anaesthetized C57BL/6 and MPO^−/−^ mice. Blood was anticoagulated with Li-heparin and erythrocytes were removed by hypotonic shock using a 155 mM ammonium chloride buffer. Remaining leukocytes were stained with rat anti-mouse Gr1 PE-Cy7 (Pharmingen, San Diego, CA, USA) and mouse anti-mouse MPO FITC (Nordic Biosite, Täby, Sweden). To block unspecific binding of the antibodies, an unlabeled rat anti-mouse Fc*γ*II/III antibody (Pharmingen) was added prior to the specific antibodies. Relevant fluorescent isotypic control antibodies, rat IgG2b PY-Cy7 (Pharmingen) and mouse IgG1 FITC (Pharmingen), were used in separate PBL samples to monitor unspecific binding. Flow cytometry was performed using a FACSCalibur cytometer with an argon laser emitting light at 488 nm (Becton Dickinson, Stockholm, Sweden). From each sample, 5000 cells were acquired and stored in ListMode data files using CellQuest software (Becton Dickinson). The data were analysed using WinList software (Becton Dickinson) and are expressed as mean ± SD percentage of positive cells (above isotypic control) of three mice per group.

### 2.3. Solutions

The composition of the physiological salt solution (PSS) used was (in mM) as follows: NaCl 119; KCl 4.7; KH_2_PO_4_ 1.18; MgSO_4_ 1.17; NaHCO_3_ 25; CaCl_2_ 2.5; glucose 5.5; EDTA 0.026. In calcium-free PSS, CaCl_2_ was omitted. High-potassium solution was PSS, where NaCl was substituted with KCl on an equimolar basis. Solutions were equilibrated with 5% CO_2_ in O_2_ and maintained at 37°C. Drugs were obtained from Sigma.

### 2.4. Statistics

Statistics was by means of unpaired Student's *t*-test, where two groups were compared, and analysis of variance when comparing a greater number of groups, using GraphPad Prism 4.02 (GraphPad Software, La Jolla, CA, USA) for calculations. Concentration-response relations were analysed by nonlinear fitting to a sigmoidal dose-response relation with variable slope. Inference was based on EC_50_ values and Hill slope. Values are given as means ± SEM.

## 3. Results

**Figure 1:**
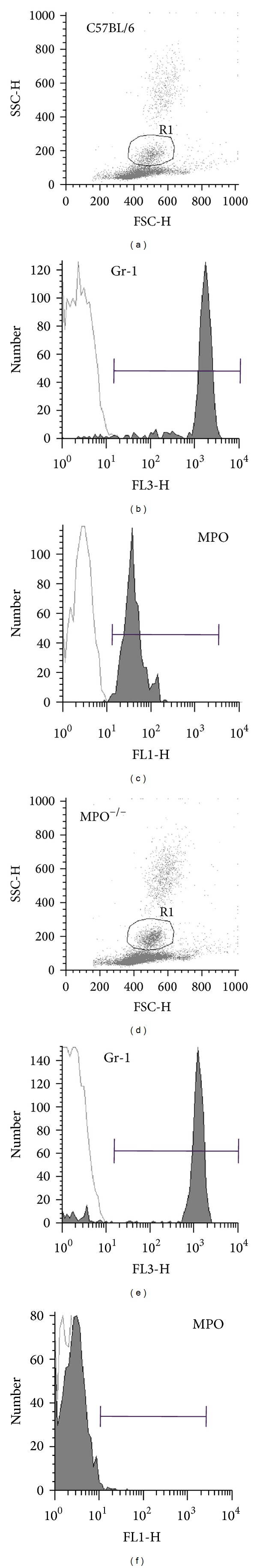
Neutrophils of MPO^−/−^ mice lack MPO expression. Representative flow cytometry data of PBL of C57BL/6 mice (a–c) and MPO^−/−^ mice (d–f). (a and d) Neutrophil gate in forward versus side scatter dot plots of C57BL/6 (a) and MPO^−/−^ mice (d). (b and e) Neutrophil expression of Gr-1 (filled peaks) as compared with isotype controls (open peaks). (c and f) Neutrophil expression of MPO (filled peaks) as compared with isotype controls (open peaks).

We used flow cytometry to assess MPO expression by peripheral blood neutrophils. As shown in Figure 1, gated neutrophils expressed high level of Gr-1 in both C57BL/6 and MPO^−/−^ mice (87.3 ± 8.3% and 94.2 ± 3.3%, in C57BL/6 and MPO^−/−^ mice, resp.) confirming that the gate indeed selected the neutrophil population. Applying the same gate, most of the C57BL/6 mice neutrophils expressed MPO (96.1 ± 2.5%) while neutrophils of MPO^−/−^ mice indeed were deficient for MPO (0.52 ± 0.51%).

**Figure 2:**
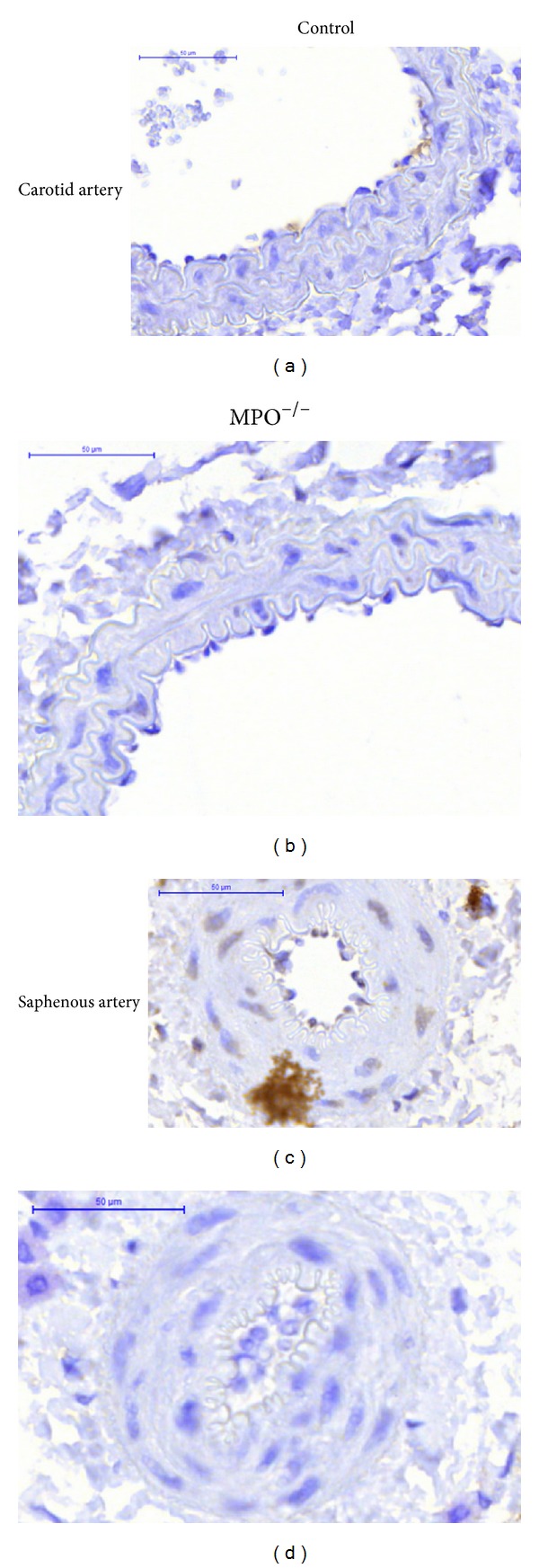
Immunohistochemical detection of myeloperoxidase in carotid (a and b) and saphenous (c and d), arteries from control (a and c) and from MPO^−/−^ (b and d) animals. Myeloperoxidase is brown-stained, seen occasionally in the endothelium and a slight staining in smooth muscle cell nuclei of distal saphenous artery in controls. Scale bar 50 *μ*m.

Immunohistochemistry (Figure 2) showed only occasional, faint staining of the endothelium in some control arteries. In distal saphenous arteries, smooth muscle cell nuclei showed slight staining in controls. No straining was seen in MPO^−/−^.

Table 1 presents body weights for the animals as well as normalized diameters for the vessels studied. No significant differences in body weights were found, and also dimensions of carotid arteries were similar between the groups. However, saphenous arteries were about 10% smaller in MPO^−/−^ animals (*P* < 0.01).

## 4. Carotid Arteries

No significant differences were found in the responses of carotid arteries to phenylephrine (1 or 3 *μ*M) or potassium either alone or in combination with 3 *μ*M PE (Table 2).

**Figure 3:**
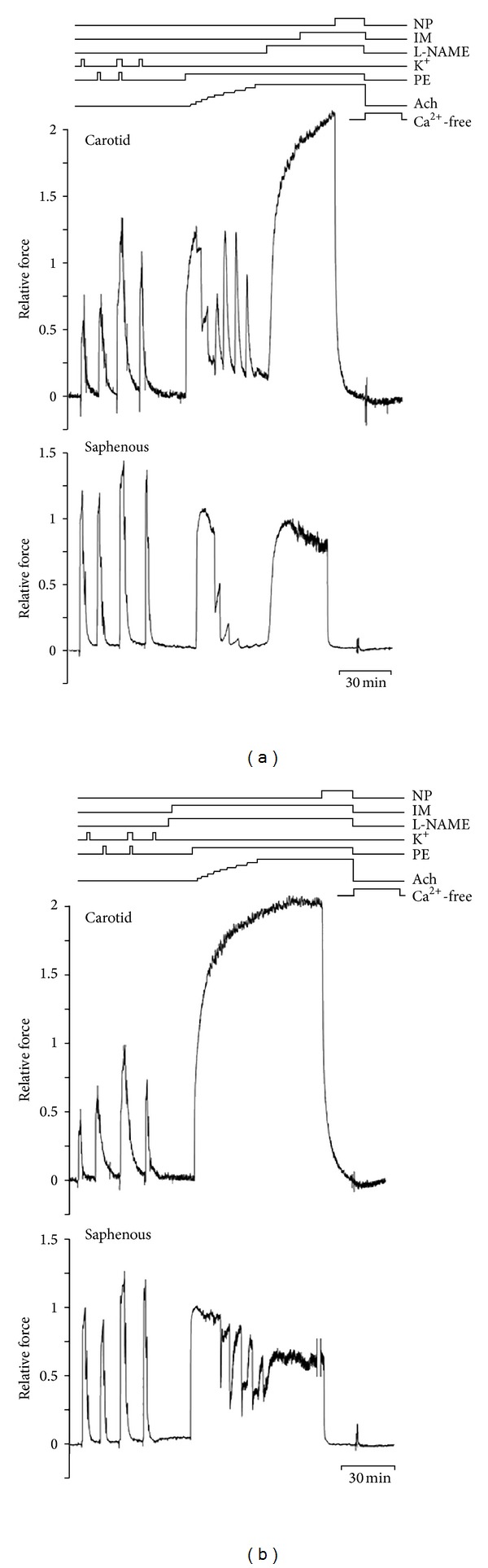
Individual experiments showing the two types of artery and the two protocols used. Original traces from carotid (upper) and saphenous (lower) arteries. In protocol in (a), a concentration-response experiment to acetylcholine was performed before addition of L-NAME and indomethacin; in (b), L-NAME and indomethacin were given before acetylcholine. Note remaining response to acetylcholine in the presence of L-NAME and indomethacin in saphenous arteries (panel (b), lower). NP: nitroprusside, IM: indomethacin, PE: phenylephrine, and Ach: acetylcholine (from 1 nM to 10 *μ*M in half-log steps).

**Figure 4:**
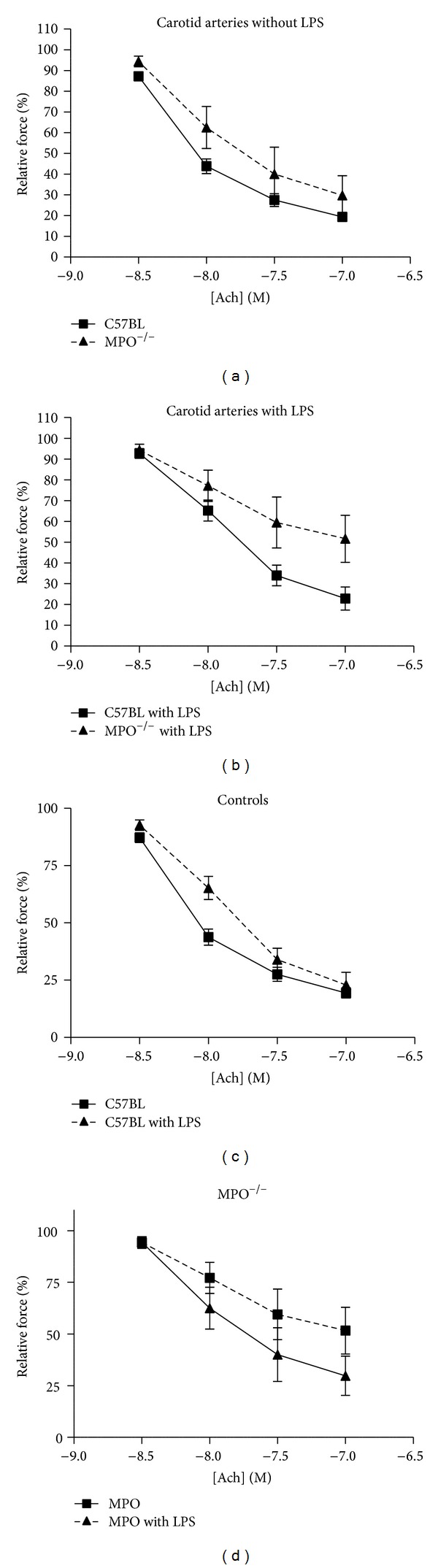
Concentration-response relation for Ach in carotid arteries from untreated animals (a) and from animals treated with lipopolysaccharide (b). Data replotted in (c) for controls and in (d) for MPO^−/−^. Response at maximal relaxation in (b) controls 21 ± 6%, MPO^−/−^  50 ± 13% (*P* < 0.01).

Arteries were activated with 1 *μ*M PE and subsequently acetylcholine was added cumulatively. Increasing concentrations of Ach caused concentration-dependent vasodilatation, but in response to concentrations of 1 *μ*M and higher also transient contractions (Figure 3(a) upper panel). These contractions were not seen in the presence of indomethacin (data not shown). The relaxing response to applied Ach was not significantly different in MPO^−/−^ and wild-type carotid arteries (Figure 4(a), *P* = 0.11).

In the continued presence of the highest concentration of Ach (10 *μ*M), L-NAME and indomethacin were added. This caused a rapid contraction in carotid arteries, which clearly exceeded that evoked by 1 *μ*M PE in the absence of L-NAME. All contractions could be reversed by addition of sodium nitroprusside in both groups (Figure 3(a) upper panel).

If the combination of L-NAME and indomethacin was added before the concentration-response curve, Ach did not cause any dilatation in carotid arteries (Figure 3(b) upper panel).

In comparison to vessels from nontreated animals, vessels taken from LPS-treated animals tended to relax less in response to Ach; this effect of LPS was most pronounced in vessels from MPO^−/−^ animals (Figure 4). Thus, after LPS treatment, carotid arteries from MPO^−/−^ animals showed a significantly smaller response to Ach than arteries from controls (Figure 4(b), *P* < 0.01).

**Figure 5:**
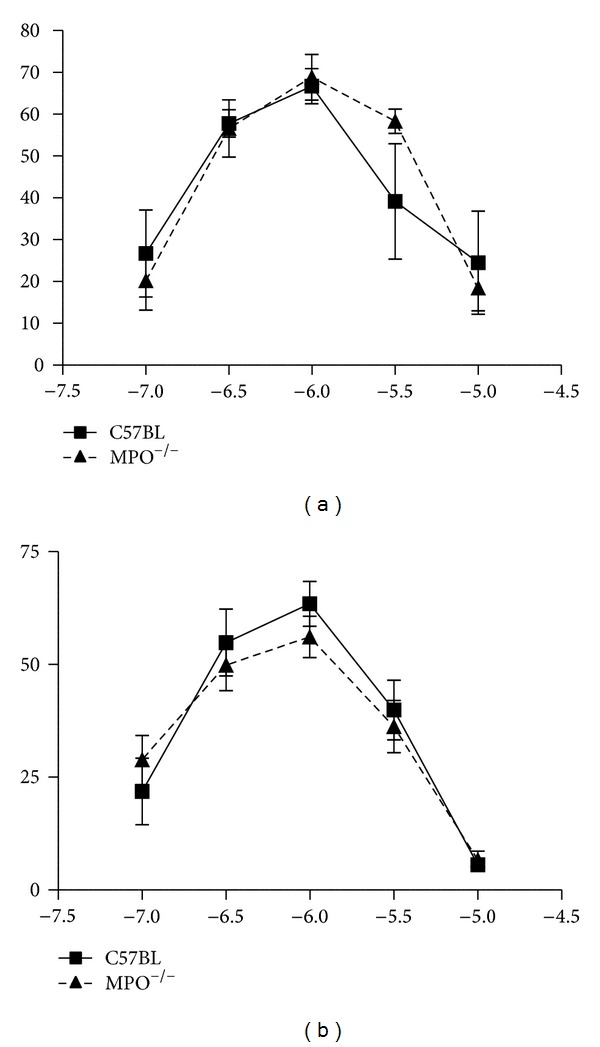
The contractile response to higher concentrations of Ach of carotid arteries from untreated (a) and LPS-treated (b) animals. No differences were seen.

The contraction in response to higher concentration of Ach seen in the carotid arteries was similar in the two groups of animals, whether or not these had been treated with LPS (Figure 5).

## 5. Saphenous Arteries

Table 3 shows responses to PE and high potassium of saphenous arteries. Responses to high potassium either alone or in combination with 3 *μ*M PE were significantly smaller in the MPO^−/−^ group (*P* < 0.01). Responses to PE were not different with the exception of the response to 1 *μ*M PE in the presence of L-NAME, which also was reduced in MPO^−/−^ (*P* < 0.01).

Saphenous arteries responded with only dilatation to all concentrations of added Ach (Figure 3(a), lower panel). As in the carotid arteries, application of L-NAME and indomethacin in the continued presence of PE and Ach caused maintained contraction. In these arteries, the contraction after application of L-NAME did not exceed that to 1 *μ*M PE alone, in contrast to the carotid arteries. Also in the saphenous arteries contractions were reversed by addition of sodium nitroprusside.

If the combination of L-NAME and indomethacin was added before the concentration-response curve, a dilator response to Ach remained, although of lower amplitude and more transient than in the absence of the inhibitors (Figure 3(b), lower panel). This response was considered to be due to EDHF. Thus, in contrast to the carotid arteries, the Ach response of the saphenous arteries appeared to consist of both L-NAME-inhibited and L-NAME-resistant components.

**Figure 6:**
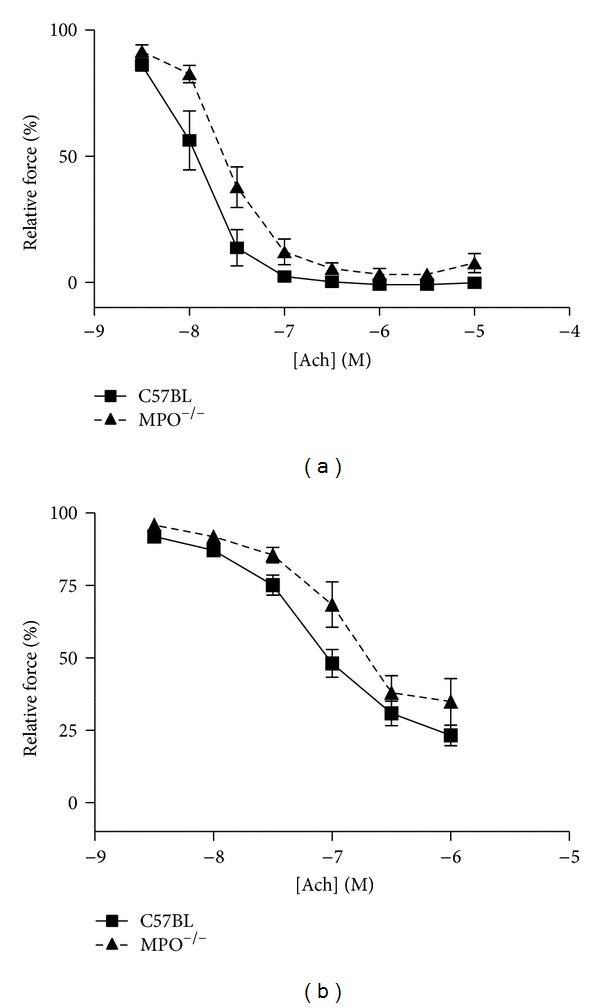
Concentration-response relation for Ach in saphenous arteries in the absence (a) and presence (b) of L-NAME and indomethacin (showing the EDHF response). Vessels from MPO^−/−^ responded less than vessels from wild-type: EC_50_ was in (a) control −7.95 ± 0.045, MPO^−/−^  −7.65 ± 0.041 (*P* < 0.001) and in (b) control −7.19 ± 0.100, MPO^−/−^  −6.98 ± 0.16 (*P* < 0.001).

The concentration-response relation to Ach (in the absence of L-NAME and indomethacin) was right-shifted in vessels from MPO^−/−^ mice in comparison to control vessels (Figure 6(a)). The same was observed after inhibition with L-NAME and indomethacin (Figure 6(b)).

Saphenous arteries from LPS-treated animals were too constricted to permit successful mounting in most cases; therefore, no data are given here.

## 6. Discussion

This study was motivated by recent interest in the role of myeloperoxidase in the development of endothelial dysfunction. The hypothesis is that the inflammatory response to accumulation and oxidation of lipids in the vascular wall will attract neutrophils, which secrete myeloperoxidase. Liberated MPO may penetrate the endothelium and accumulate extracellularly in the subintimal space, where the activity of MPO will reduce the half-life of nitric oxide, released from the endothelium, and thus counteract NO-dependent vasodilator mechanisms and contribute to endothelial dysfunction. Alternatively products from MPO activity could cause long-lasting damage to the endothelium after initial, transient contacts between neutrophils and endothelial cells.

Toward this background, it was hypothesized that mature animals lacking MPO would show greater NO-dependent relaxation than their normal counterparts, since in the latter group the action of NO would constantly be antagonised by the action of MPO. It was also hypothesized that the MPO^−/−^ animals would be less susceptible to endothelial dysfunction if challenged by LPS. Neither hypothesis could be confirmed in the current study. Thus in carotid arteries, where the Ach response seemed completely dependent on NO (fully blocked by L-NAME), responses to Ach were similar in MPO^−/−^ and controls.

In MPO^−/−^, the saphenous arteries were smaller and had reduced contractile responses to potassium. The latter observation could be secondary to their smaller size, but the reduction in force is proportionately greater than the reduction in size, suggesting also other reasons for the loss of force production. Whether this is related to the lack of MPO remains to be determined. In the saphenous arteries from controls, L-NAME enhanced the response to phenylephrine in contrast to saphenous arteries from MPO^−/−^ and to carotid arteries of either type. This indicates a lesser NO influence and increased endothelial dysfunction in saphenous arteries from MPO^−/−^, which parallels the results of Brennan et al. [12] of increased tendency for atherosclerosis. After NO synthesis inhibition with L-NAME and cyclooxygenase inhibition with indomethacin, the relaxation to Ach was reduced in MPO^−/−^ compared to controls, which suggest an altered EDHF response as well in the knockout animals. It is possible that the EDHF deficiency may in some way relate to the abnormality in the potassium response. Since this endothelial dysfunction was found only in saphenous and not carotid arteries, which differ in respect to the EDHF component, it is possible that the influence of MPO deficiency on endothelial function may primarily relate to the EDHF response.

Eiserich et al. [11] studied isolated aorta from younger mice before and after treatment with a higher dose of LPS for shorter time (12.5 mg/kg for 4 h). They reported very similar concentration-response relations in untreated animals. After LPS treatment, no change in the Ach sensitivity in MPO^−/−^ mice was observed, but a reduced sensitivity in wild-type animals. That observation would be consistent with MPO released from neutrophils during the inflammatory reaction to LPS and accumulating in the vessel wall, reducing the effect of NO liberated from the endothelium. However, our current data do not support such a scheme. Indeed, in the present experiments, carotid arteries from MPO^−/−^ were more vulnerable to LPS treatment than controls, insofar as relaxation to Ach was impaired in these vessels and was little affected in controls. Whether this can be attributed to a different time course of the inflammatory process in the endothelium of these vessels is not known. Although the present study used a different protocol for LPS treatment, this has previously been used successfully to reduce endothelial function in mice [14]. The differences between the studies can obviously also be attributed to gender and age differences. Further studies will be needed to clarify this point.

In response to high concentrations of acetylcholine, contractions rather than relaxations were obtained. These likely correspond to the endothelium-dependent contractile factor (EDCF) that has been demonstrated in other blood vessels [15], where it has been shown to be secondary to production of prostanoids, in particular thromboxane [16]. Constituent with this, the contractions were inhibited by indomethacin in the present experiments. Their nature was not further analysed, but also the EDCF component of the Ach response was unaltered by MPO knockout.

To verify the knockout of the MPO gene product, we used FACS analysis to show elimination of MPO from neutrophilic leukocytes, with unchanged levels of peroxidase activity in eosinophils. This verifies the specificity for neutrophils of MPO in white blood cells. Immunohistochemistry showed faint staining for MPO in the vessel walls from control animals, although transient contacts between white blood cells and endothelium are difficult to demonstrate with this technique.

## 7. Conclusion

The present study indicates that endothelial function in aged MPO^−/−^ mice is not significantly different from control. If MPO plays a significant role in degradation of NO, one might expect this system to be affected by life-long enhancement of NO effects. This does not seem to be the case here, although we cannot exclude that altered sensitivity to NO and an altered EDHF response are adaptive changes in response to altered NO levels.

## Figures and Tables

**Table 1:** Mouse body weight and diameter of carotid and saphenous arteries in experimental groups.

	MPO^−/−^		Wild-type	
	Control	LPS-treated	Control	LPS-treated
	M ± SEM	M ± SEM	M ± SEM	M ± SEM
Body weight, g	25.80 ± 1.10 *n* = 5	22.88 ± 1.21 *n* = 7	25.05 ± 0.83 *n* = 5	22.72 ± 0.35 *n* = 7
*d*_100_ carotid, *μ*m	518.79 ± 13.13 *n* = 10	514.09 ± 4.98 *n* = 10	518.70 ± 8.47 *n* = 8	529.62 ± 18.15 *n* = 10
*d*_100_ saphenous, *μ*m	223.49 ± 6.03** *n* = 10		254.34 ± 7.00 *n* = 10	

**P < 0.01.

**Table 2:** General data for carotid arteries in experimental groups.

Carotid arteries	MPO^−/−^		Wild-type	
	Control	LPS-treated	Control	LPS-treated
	M ± SEM mN/mm	M ± SEM mN/mm	M ± SEM mN/mm	M ± SEM mN/mm
Contraction (tension)				
1 *μ*M PE	1.31 ± 0.21 *n* = 5	1.46 ± 0.08 *n* = 8	1.51 ± 0.06 *n* = 4	1.52 ± 0.09 *n* = 8
1 *μ*M PE after L-NAME	1.43 ± 0.10 *n* = 5	1.37 ± 0.04 *n* = 2	1.56 ± 0.13 *n* = 4	1.64 ± 0.15 *n* = 2
3 *μ*M PE	0.90 ± 0.11 *n* = 10	1.13 ± 0.06 *n* = 10	1.03 ± 0.07 *n* = 8	1.21 ± 0.06 *n* = 10
3 *μ*M PE in high K^+^	1.44 ± 0.11 *n* = 10	1.56 ± 0.06 *n* = 10	1.54 ± 0.11 *n* = 8	1.72 ± 0.11 *n* = 10
High K^+^	1.22 ± 0.13 *n* = 10	1.41 ± 0.09 *n* = 10	1.27 ± 0.11 *n* = 8	1.40 ± 0.09 *n* = 10
Relaxation (% of change in tension)				
Maximal relaxation to acetylcholine	92.20 ± 1.15 *n* = 5	93.86 ± 3.88 *n* = 8	94.24 ± 1.36 *n* = 4	94.90 ± 1.65 *n* = 8

**Table 3:** General data for saphenous arteries in experimental groups.

Saphenous arteries	MPO^−/−^	Wild-type
	Control	Control
	M ± SEM mN/mm	M ± SEM mN/mm
Contraction (tension)		
1 *μ*M PE	2.69 ± 0.41 *n* = 5	2.68 ± 0.40 *n* = 5
1 *μ*M PE after L-NAME	2.91 ± 0.15** *n* = 5	3.59 ± 0.11 *n* = 5
3 *μ*M PE	2.90 ± 0.20 *n* = 10	3.12 ± 0.17 *n* = 10
3 *μ*M PE in high K^+^	3.61 ± 0.21** *n* = 10	4.36 ± 0.09 *n* = 10
High K^+^	3.36 ± 0.21** *n* = 10	4.03 ± 0.08 *n* = 10
Relaxation (% of change in tension)		
Maximal relaxation to acetylcholine	99.07 ± 0.81 *n* = 5	99.89 ± 0.12 *n* = 5
Maximal relaxation to acetylcholine after L-NAME	94.37 ± 1.60 *n* = 5	97.08 ± 0.50 *n* = 5

**P < 0.01.
